# Inhibition of ferroptosis by POLE2 in gastric cancer cells involves the activation of NRF2/GPX4 pathway

**DOI:** 10.1111/jcmm.17983

**Published:** 2023-12-09

**Authors:** Hui Jian, Zhi‐Qiang Chen, Heng Du, Ting Liao, Yi‐Chen Sun, Dong Ke, Yang Yu

**Affiliations:** ^1^ Department of Gastrointestinal Surgery Affiliated Hospital of Jianghan University Wuhan Hubei China; ^2^ Department of Gastrointestinal Surgery Huanggang Central Hospital Affiliated to Yangtze University Huanggang Hubei China; ^3^ Department of Gastroenterology Affiliated Hospital of Jianghan University Wuhan Hubei China; ^4^ Department of Oncology Affiliated Hospital of Jianghan University Wuhan Hubei China; ^5^ Department of Gastrointestinal Surgery Renmin Hospital of Wuhan University Wuhan Hubei China

**Keywords:** ferroptosis, gastric cancer, GPX4, POLE2

## Abstract

Gastric cancer results in great cancer mortality worldwide, and inducing ferroptosis dramatically improves the malignant phenotypes of gastric cancer. DNA polymerase epsilon subunit 2 (POLE2) plays indispensable roles in tumorigenesis; however, its involvement and molecular basis in ferroptosis and gastric cancer are not clear. Human gastric cancer cells were infected with lentiviral vectors to knock down or overexpress POLE2, and cell ferroptosis was detected. To further validate the involvement of nuclear factor erythroid 2‐related factor 2 (NRF2) and glutathione peroxidase 4 (GPX4), lentiviral vectors were used. POLE2 expression was elevated in human gastric cancer cells and tissues and closely correlated with clinicopathological features in gastric cancer patients. POLE2 knockdown was induced, while POLE2 overexpression inhibited ferroptosis of human gastric cancer cells, thereby modulating the malignant phenotypes of gastric cancer. Mechanistic studies revealed that POLE2 overexpression elevated NRF2 expression and activity and subsequently activated GPX4, which then prevented lipid peroxidation and ferroptosis in human gastric cancer cells. In contrast, either NRF2 or GPX4 silence significantly prevented POLE2 overexpression‐mediated inductions of cell proliferation, migration, invasion and inhibition of ferroptosis. POLE2 overexpression inhibits ferroptosis in human gastric cancer cells through activating NRF2/GPX4 pathway, and inhibiting POLE2 may be a crucial strategy to treat gastric cancer.

## 1 INTRODUCTION

Gastric cancer, one of the most prevalent tumours in the digestive system, results in great cancer mortality worldwide, and the currently available treatments are barely satisfactory.^1^, ^2^, ^3^, ^4^ Ferroptosis is a novel regulated cell death mainly featured as iron‐associated accumulation of lipid reactive oxygen species (ROS) that is produced through a Fenton reaction involving ferrous iron.^5^, ^6^, ^7^, ^8^, ^9^ Excessive lipid ROS then facilitates the oxidation of polyunsaturated fatty acids (PUFAs) forms lipid hydroperoxides (L‐OOH) and hydroxyeicosatetraenoic acids (HETEs).^10^, ^11^ Glutathione peroxidase 4 (GPX4), a glutathione (GSH)‐relied selenoenzyme, reduces toxic L‐OOH to nontoxic lipid alcohols and thus prevents ferroptotic cell death.^12^, ^13^ Nuclear factor erythroid 2‐related factor 2 (NRF2) acts as a transcriptional factor to upregulate the expression of multiple antioxidant genes, including GPX4, upon oxidative stress (e.g., ferroptosis).^14^, ^15^, ^16^ Increasing evidences have demonstrated an involvement of ferroptosis in tumour progression, and inducing ferroptosis dramatically improves the malignant phenotypes of gastric cancer.^17^, ^18^ Yang et al.^19^ recently demonstrated that inhibiting ferroptosis through induction of GSH synthesis significantly facilitated the progression of gastric cancer. In contrast, enhancing ferroptotic cell death suppressed the growth of gastric cancer cells, thereby improving the prognosis of gastric cancer.^20^ Meanwhile, promoting ferroptosis also sensitised gastric cancer cells to the chemotherapy of 5‐fluorouracil.^21^ Therefore, targeting ferroptosis may be a potential strategy to treat gastric cancer.

DNA polymerase epsilon subunit 2 (POLE2) is primarily involved in DNA replication and repair, while recent studies have reported indispensable roles of POLE2 in tumorigenesis.^22^ Li et al.^23^ found that knockdown of POLE2 could suppress proliferation but initiate apoptosis of lung adenocarcinoma cells. Meanwhile, POLE2 knockdown also blocked proliferation, migration and induced apoptosis of oesophageal squamous cells.^24^ Consistently, Zhang et al. previously demonstrated that POLE2 expression was elevated in renal cell carcinoma (RCC) tissues and that higher POLE2 level correlated with poor prognosis. Moreover, POLE2 silence could inhibit proliferation, migration and facilitate apoptosis of RCC cells, thereby attenuating RCC tumorigenesis and growth in vitro.^25^ A very recent study also identified an abnormal elevation of POLE2 in human glioblastoma tissues, and they determined that knocking down POLE2 inhibited the malignancies of glioblastoma.^26^ In addition to the well‐known oncogenic role, POLE2 protein level was also regulated by iron metabolism, and proper iron supplementation could restore POLE2 expression.^27^ Herein, we intend to determine the involvement and mechanism of POLE2 in ferroptosis of gastric cancer.

## 2 MATERIALS AND METHODS

### 2.1 Cell culture

Human gastric cancer cells (AGS, BGC‐823, HGC‐27, MGC‐803, MKN‐28, MKN‐45, MKN‐74 and SGC‐7901) and normal human gastric epithelium cells (GES‐1) were cultured as previously described.^28^ To stimulate ferroptosis, MGC‐803 and SGC‐7901 cells were treated with 5 μmol/L erastin (S7242; Selleck Chemicals), 2 μmol/L RSL3 (S8155; Selleck Chemicals) or dimethyl sulfoxide (DMSO, D2650; Sigma‐Aldrich) for 72 h.^29^ To knock down or overexpress POLE2, MGC‐803 and SGC‐7901 cells were infected with lentiviral vectors containing the short hairpin RNA against POLE2 (shPOLE2), full‐length human POLE2 sequence (NM_001197330.2) or respective controls (shCtrl for shPOLE2 and Ctrl for POLE2) at a multiplicity of infection (MOI) of 10. Four hours later, the medium was aspirated and replaced with fresh medium containing 10% foetal bovine serum (FBS) for an additional 72 h. The lentiviral vectors were generated by Shanghai Yibeirui Biomedical Technology Co., Ltd., and the target sequences were provided as follows: 5′‐CGATTGTTCTTGGAATGATA‐3′ (shPOLE2#1) and 5′‐CGTGAAGACTTAGTAAATAA‐3′ (shPOLE2#2). To inhibit ferroptosis, 1 μmol/L ferrostain‐1 (Fer‐1, S7243; Selleck Chemicals) or 0.2 μmol/L liproxstatin‐1 (Lip‐1, S7699; Selleck Chemicals) was added to the medium for 72 h after the aspiration of shPOLE2.^29^ To knock down NRF2 or GPX4, cells were pre‐infected with shNRF2 (sc‐37,030; Santa Cruz) or shGPX4 (sc‐44465; Santa Cruz) at a MOI of 10 for 4 h and then cultured in fresh medium containing 10% FBS for an additional 24 h before POLE2 overexpression.

### 2.2 Quantitative real‐time PCR

Cells were collected using TRIzol (15596018; Thermo Fisher Scientific) for total RNA extraction, and RNA concentration as well as quality were determined by Nanodrop 2000c spectrophotometer (Thermo Fisher Scientific).^30^, ^31^, ^32^, ^33^, ^34^ Next, cDNA was synthesized using HiScript® Q RT SuperMix for qPCR (+gDNA wiper) (R123‐01; Vazyme Biotech Co., Ltd.) following the manufacturer's instructions, and then quantitative real‐time PCR was performed with AceQ® qPCR SYBR Green Master Mix (Q111‐02; Vazyme Biotech Co., Ltd.) on an ABI 7300 PCR Detection System (Applied Biosystems). The primer sequences were provided as follows: POLE2 forward 5′‐TGAGAAGCAACCCTTGTCATC‐3′, POLE2 reverse 5′‐TCATCAACAGACTGACTGCATTC‐3′; NRF2 forward 5′‐TCCAGTCAGAAACCAGTGGAT‐3′, NRF2 reverse 5′‐GAATGTCTGCGCCAAAAGCTG‐3′; Glyceraldehyde‐3‐phosphate dehydrogenase (GAPDH) forward 5′‐CGGATTTGGTCGTATTGGG‐3′, GAPDH reverse 5′‐GATTTTGGAGGGATCTCGC‐3′.

### 2.3 Western blot

Cells were lysed with RIPA lysis buffer (P0013B; Beyotime Biotechnology) for total protein extraction, and protein concentration was determined by a Pierce BCA Protein Assay Kit (23227; Thermo Fisher Scientific).^35^, ^36^, ^37^, ^38^ Next, total proteins were separated by sodium dodecyl sulphate‐polyacrylamide gel electrophoresis and transferred onto polyvinylidene difluoride membranes, which were then incubated overnight at 4°C with anti‐POLE2 (sc‐398582; Santa Cruz), anti‐GPX4 (ab125066; Abcam), anti‐NRF2 (ab62352; Abcam) or anti‐GAPDH (ab8245; Abcam) after blocking with 5% skim milk. On the second day, membranes were rinsed with Tris‐based saline‐Tween 20 for three times and incubated with horseradish peroxidase (HRP)‐conjugated secondary antibodies for 2 h at room temperature. Subsequently, proteins were visualised using BeyoECL Plus reagent (P0018; Beyotime Biotechnology) and analysed by Image Lab Version 6.0 software (Bio‐Rad, Hercules).

### 2.4 Cell viability

Cells in 96‐well plates were incubated with 10 μL Cell Counting Kit‐8 (CCK‐8, C0037; Beyotime Biotechnology) for 4 h at 37°C, and the optical density at 450 nm (OD450) was measured by a Synergy HT microplate reader (Bio‐Tek).^39^, ^40^

### 2.5 Transwell assay

Transwell assay was performed to evaluate cell migration and invasion using a CytoSelect™ 24‐Well Cell Migration and Invasion Assay Combo Kit (CBA‐100; Cell Biolabs) following the manufacturer's instructions. Briefly, cells were resuspended and placed in the upper chamber, while fresh medium containing 30% FBS was added to the lower chamber. Twenty‐four hours later, cells remaining on the upper surface were removed, and then the inserts were washed and incubated with 200 μL extraction solution for 10 min, which was then measured at 560 nm by a Synergy HT microplate reader. For the analysis of cell invasion, the inserts were coated with a uniform layer of dried basement membrane matrix solution.

### 2.6 Lactate dehydrogenase (LDH) release

LDH release was measured to evaluate cell death using a LDH‐Cytotoxicity Assay Kit (ab65393; Abcam) following the manufacturer's instructions.^41^, ^42^, ^43^ Briefly, cell medium was collected and centrifuged at 600 *g* for 10 min to obtain clear medium solution, which was then incubated with the WST substrate mix and LDH assay buffer for 30 min at room temperature. Next, the absorbance at 450 nm with a reference wavelength at 650 nm was measured by a Synergy HT microplate reader.

### 2.7 Analysis of ferrous iron

Ferrous iron level was measured using an Iron Assay Kit (ab83366; Abcam) following the manufacturer's instructions. Briefly, cells were homogenised using iron assay buffer and centrifuged at 16000 *g* for 10 min to obtain cell‐free supernatants, which were then incubated with 5 μL assay buffer at 37°C for 30 min, followed by the incubation with 100 μL iron probe at 37°C for 1 h. Next, OD593 was measured by a Synergy HT microplate reader.

### 2.8 Analysis of ROS and lipid peroxidation

Reactive oxygen species was measured using a 6‐carboxy‐2′,7′‐dichlorodihydrofluorescein diacetate (H2DCFDA, C2938; Thermo Fisher Scientific) as previously described.^44^, ^45^, ^46^ Briefly, cells were incubated with 5 μmol/L H2DCFDA probe at 37°C for 1 h, and then the fluorescence was measured with an excitation wavelength of 488 nm and emission at 525 nm by a spectrofluorometer. To analyse lipid peroxidation, malondialdehyde (MDA) level was measured using a Lipid Peroxidation Assay Kit (ab118970; Abcam) following the manufacturer's instructions. Briefly, cells were homogenised using Lysis Buffer and centrifuged at 13000 *g* for 10 min to obtain cell‐free supernatants, which were then incubated with 600 μL of TBA reagent at 95°C for 1 h. Next, the reaction mix containing MDA‐TBA adduct was measured at 532 nm by a Synergy HT microplate reader.

### 2.9 12/15‐HETE assay

12/15‐HETE levels in cell medium were evaluated using 12/15‐HETE ELISA kits (ab133034/ab133035; Abcam) following the manufacturer's instructions.

### 2.10 Measurements of GSH level and GPX4 activity

Glutathione level was measured using a GSH Assay Kit (ab239727; Abcam) following the manufacturer's instructions. Briefly, cells were homogenised with 5% sulfosalicylic acid solution and centrifuged to obtain the cell‐free supernatants, which were then incubated with Substrate Mix A, Substrate Mix B, Enzyme Mix A, Enzyme Mix B, Enzyme Mix C and GSH assay buffer. Next, the absorbance was measured at 450 nm by a Synergy HT microplate reader. GPX4 activity was measured by LC–MS as previously described by synthesizing a methanolic phosphatidylcholine hydroperoxide.^47^

### 2.11 Analysis of NRF2 transcriptional activity

NRF2 transcriptional activity was measured using a TransAM® NRF2 Kit (50296; Active Motif) following the manufacturer's instructions. Briefly, cell nuclear extracts were prepared and diluted in Complete Lysis buffer and then incubated at room temperature for 1 h in a 96‐well plate on which has been immobilised oligonucleotide containing the ARE consensus binding site of NRF2. After being washed, the plate was added with anti‐NRF2 antibody and incubated at room temperature for 1 h, followed by an incubation with HRP‐conjugated secondary antibody at room temperature for an additional 1 h. Then, the developing solution was added to the plate, incubated at room temperature for 10 min and then detected at 450 nm with a reference wavelength of 655 nm by a spectrofluorometer.

### 2.12 Clinical samples

Human gastric cancer and adjacent normal tissues were obtained from the Affiliated Hospital of Jianghan University according to the guidelines of the Declaration of Helsinki, and written informed consents were obtained from all patients. Tissues were immediately preserved in liquid nitrogen, and all experimental procedures were approved by the Ethics Committee of the Affiliated Hospital of Jianghan University.

### 2.13 Statistical analysis

Results were exhibited as the mean ± standard deviation and analysed using SPSS 22.0 software. Data analysis between two groups was processed by unpaired, two‐tailed Student's *t*‐test, while comparisons among multiple groups were conducted using one‐way anova followed by the Tukey post hoc test. Two‐sided *p* value threshold was set at 0.05 to be statistically significant.

## 3 RESULTS

### 3.1 POLE2 expression is elevated in human gastric cancer

**FIGURE 1:**
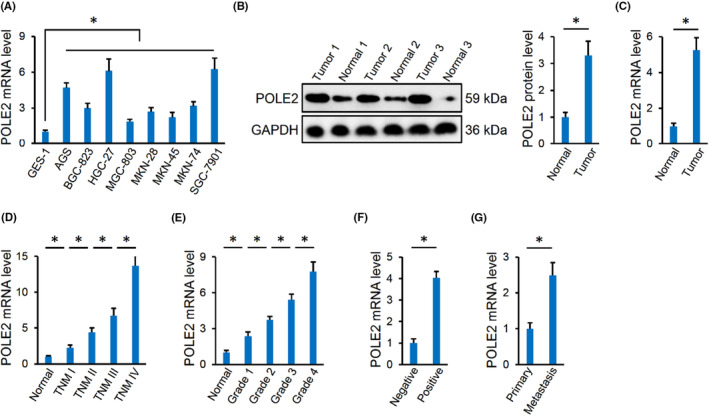
POLE2 expression is elevated in human gastric cancer. (A) POLE2 mRNA level in different human gastric cancer cells and human GES‐1. (B, C) POLE2 mRNA and protein levels in human gastric cancer tissues and adjacent normal tissues. (D, E) POLE2 mRNA level in human gastric cancer tissues from patients with different TNM stages and clinicopathological grades. (F) POLE2 mRNA level in human gastric cancer tissues from patients with or without lymph node metastasis. (G) POLE2 mRNA level in primary and metastasis human gastric cancer tissues. *n* = 6 and *p < 0.05 versus matched groups.

As indicated in Figure 1A, POLE2 mRNA level was elevated in human gastric cancer cells compared to normal human gastric epithelium cells, and POLE2 mRNA as well as protein levels were also increased in human gastric cancer tissues (Figure 1B,C). In addition, gastric cancer patients with a higher POLE2 mRNA level exhibited advanced TNM stage and clinicopathological grade (Figure 1D,E). Meanwhile, POLE2 level in gastric cancer patients with lymph node metastasis was higher than those without metastasis (Figure 1F). Moreover, we found that the metastasis lesions exhibited higher POLE2 expression than the primary tumours (Figure 1G). These results indicate that POLE2 expression is elevated in human gastric cancer, and closely associates with clinicopathological features in gastric cancer patients.

### 3.2 POLE2 knockdown induces ferroptosis of human gastric cancer cells

**FIGURE 2:**
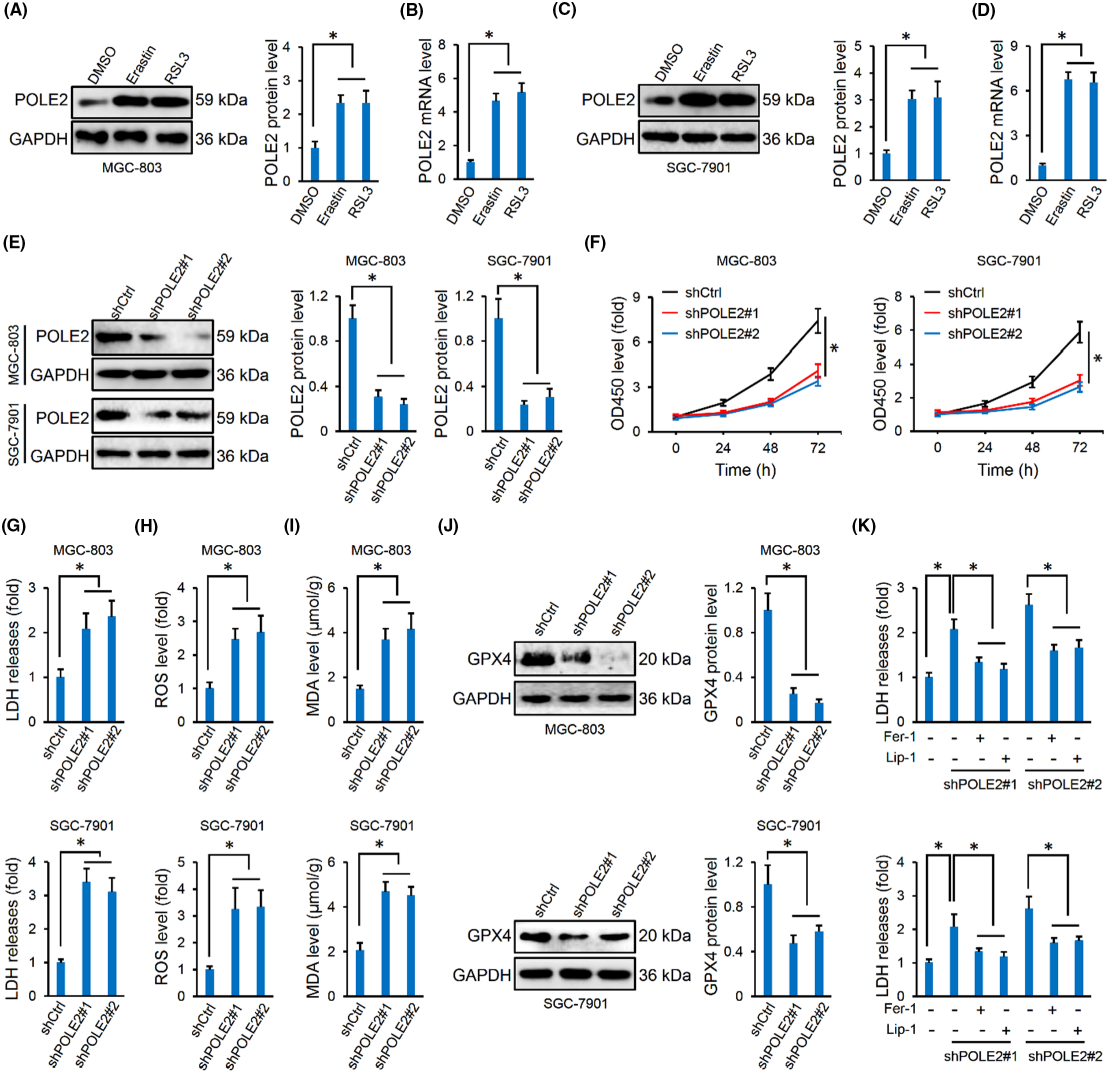
POLE2 knockdown induces ferroptosis of human gastric cancer cells. (A, B) POLE2 mRNA and protein levels in MGC‐803 cells with DMSO, erastin or RSL3 stimulations. (C, D) POLE2 mRNA and protein levels in SGC‐7901 cells with DMSO, erastin or RSL3. (E) POLE2 protein levels in MGC‐803 and SGC‐7901 cells infected with two independent shPOLE2. (F) Cell proliferation detected by the CCK‐8 method. (G) LDH level in the medium. (H, I) Intracellular ROS and MDA levels. (J) GPX4 protein levels in MGC‐803 and SGC‐7901 cells infected with two independent shPOLE2. (K) LDH level in the medium from POLE2‐silenced human gastric cancer cells with Fer‐1 or Lip‐1 treatments. *n* = 6 and *p < 0.05 versus matched groups.

To clarify the effect of POLE2 on the ferroptotic death of gastric cancer cells, the cells were stimulated with erastin or RSL3 to evaluate the alteration of POLE2 during ferroptotic cell death. As indicated in Figure 2A–D, the mRNA and protein levels of POLE2 were significantly upregulated by erastin or RSL3. Next, POLE2 expression was knocked down in these cells by lentivirus‐induced RNAi using two independent shRNA to avoid the off‐target effect (Figure 2E). Amazingly, POLE2 knockdown could suppress proliferation, migration and invasion of human gastric cancer cells (Figure 2F and Figure S1A,B). In contrast, cell damage and death were enhanced by POLE2 silence, as evidenced by increased LDH release (Figure 2G). Lipid peroxidation is an important characteristic of ferroptosis and is typically formed from the PUFAs chains of membrane lipid.^10^ As indicated in Figure 2H,I, ROS and MDA levels were elevated in POLE2‐silenced gastric cancer cells. Accordingly, POLE2 knockdown also facilitated PUFAs oxidation and membrane destruction, as evidenced by increased 12/15‐HETE (Figure S1C,D). Iron accumulation is the other feature and a prerequisite of ferroptosis; however, we found that POLE2 knockdown did not affect intracellular ferrous iron level in MGC‐803 or SGC‐7901 cells (Figure S1E). Interestingly, we found that POLE2 silence did not affect intracellular GSH level, but significantly elevated the expression and activity of GPX4, a selenoenzyme required for scavenging toxic L‐OOH and preventing ferroptosis^47^ (Figure 2J and Figure S2F,G). To further determine whether the increased death in POLE2‐silenced gastric cancer cells was ferroptosis, two different inhibitors of ferroptosis were used. As expected, both Fer‐1 and Lip‐1 effectively ameliorated POLE2 knockdown‐induced cell damage and death, indicating an involvement of ferroptosis in this process. Taken together, we speculate that POLE2 knockdown induces ferroptosis and subsequently mitigates the malignancies of human gastric cancer cells.

### 3.3 POLE2 overexpression inhibits ferroptosis of human gastric cancer cells

**FIGURE 3:**
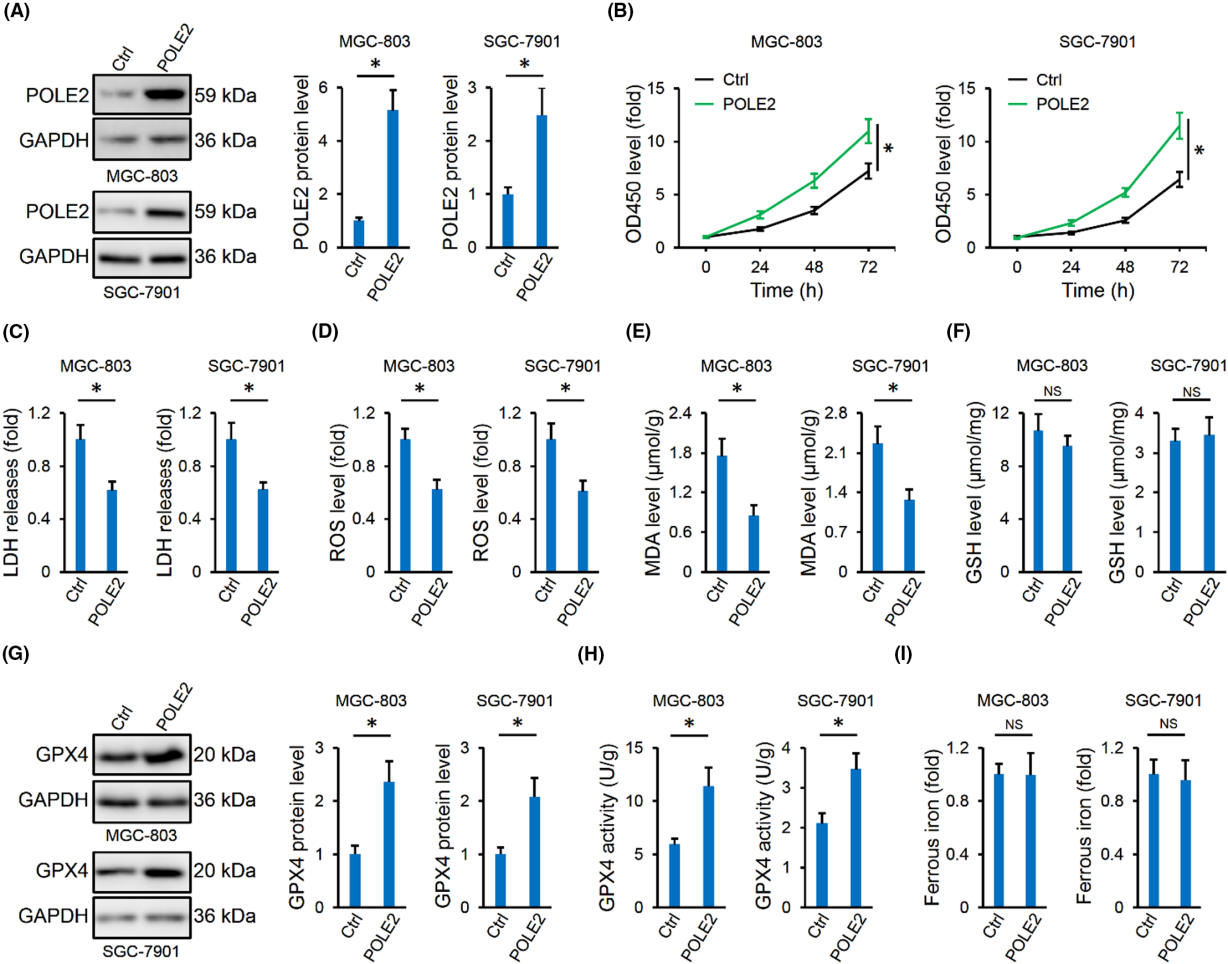
POLE2 overexpression inhibits ferroptosis of human gastric cancer cells. (A) POLE2 protein levels in MGC‐803 and SGC‐7901 cells with or without POLE2 overexpression. (B) Cell proliferation detected by a CCK‐8 method. (C) LDH level in the medium. (D, E) Intracellular ROS and MDA levels. (F) Intracellular GSH levels. (G, H) GPX4 protein and activity levels in MGC‐803 and SGC‐7901 cells with or without POLE2 overexpression. (I) Ferrous iron levels. NS indicates no significance. *n* = 6 and *p < 0.05 versus matched groups.

Conversely, MGC‐803 and SGC‐7901 cells were overexpressed with POLE2 using lentiviral vectors, and POLE2 protein level was increased in the two cells (Figure 3A). As expected, proliferative, migrative and invasive rates were enhanced by POLE2 overexpression (Figure 3B and Figure S2A,B). In contrast, cell damage and death were blocked by POLE2 overexpression (Figure 3C). As shown in Figure 3D,E and Figure S2C,D, ROS generation and lipid peroxidation were also reduced in POLE2‐overexpressed human gastric cancer cells, followed by improvements of PUFAs oxidation and membrane destruction. In line with the knockdown results, POLE2 overexpression also made no alteration on intracellular GSH level but elevated GPX4 expression and activity in the two cells (Figure 3F–H). In addition, intracellular ferrous iron level was also unaffected by POLE2 overexpression (Figure 3I). These data suggest that POLE2 overexpression inhibits ferroptosis and subsequently facilitates the malignant phenotypes of human gastric cancer cells.

### 3.4 POLE2 overexpression inhibits ferroptosis of human gastric cancer cells through activating NRF2/GPX4 pathway

**FIGURE 4:**
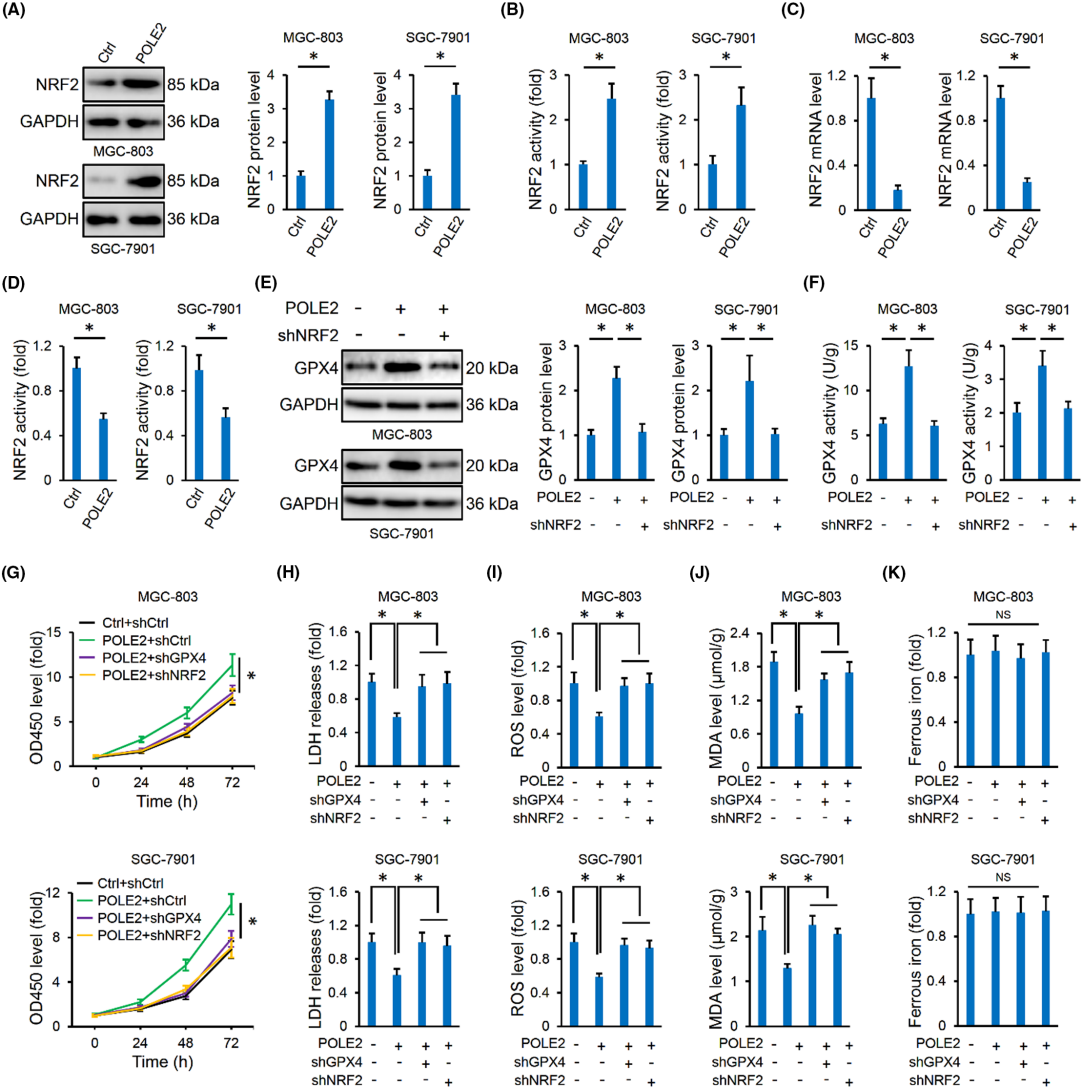
POLE2 overexpression inhibits ferroptosis of human gastric cancer cells through activating the NRF2/GPX4 pathway. (A, B) NRF2 protein and activity levels in MGC‐803 and SGC‐7901 cells with or without POLE2 overexpression. (C, D) NRF2 mRNA and activity levels in MGC‐803 and SGC‐7901 cells with or without NRF2 silence. (E, F) GPX4 protein and activity levels in POLE2‐overexpressed MGC‐803 and SGC‐7901 cells with or without NRF2 silence. (G) Cell proliferation was detected by the CCK‐8 method. (H) LDH level in the medium. (I, J) Intracellular ROS and MDA levels. (K) Ferrous iron levels. NS indicates no significance. *n* = 6 and *p < 0.05 versus matched groups.

The aforementioned data indicated that POLE2 knockdown decreased, while POLE2 overexpression increased GPX4 expression and activity, and NRF2 is a critical transcriptional factor to elevate the level of antioxidant genes, including GPX4 during ferroptosis.^48^ We thus determined whether the NRF2/GPX4 pathway was essential for POLE2 overexpression‐mediated inhibition on ferroptosis in gastric cancer. As indicated in Figure 4A,B, POLE2 overexpression significantly increased NRF2 expression and activity in the two gastric cancer cells. And NRF2 silence blocked POLE2 overexpression‐mediated induction of GPX4 expression and activity (Figure 4C–F). To further clarify the involvement of NRF2/GPX4 pathway, POLE2‐overexpressed cells were knocked down with NRF2 or GPX4, respectively. As shown in Figure 4G and Figure S3A,B, either NRF2 or GPX4 silence significantly prevented POLE2 overexpression‐mediated cell malignancies. In addition, the improvement of cell death by POLE2 overexpression was also blocked in the absence of NRF2 or GPX4, as evidenced by increased LDH release (Figure 4H). As expected, the inhibitory effects of ROS generation, lipid peroxidation and PUFAs oxidation in human gastric cancer cells with POLE2 overexpression were also blunted by either NRF2 or GPX4 silence (Figure 4I,J and Figure S3C,D). Yet, neither NRF2 nor GPX4 silence affected intracellular iron levels in POLE2‐overexpressed cells (Figure 4K). Collectively, these results imply that POLE2 overexpression inhibits ferroptosis of human gastric cancer cells through activating the NRF2/GPX4 pathway.

## 4 DISCUSSION

Gastric cancer is one of the most prevalent tumours in the digestive system and causes great cancer‐related mortality worldwide. Despite the advances in the diagnosis and treatment, the prognosis of gastric cancer remains poor.^1^ Therefore, identifying the underlying molecular basis is vital to establishing effective target therapy. In this study, POLE2 expression was found to be elevated in gastric cancer and closely correlated with the clinicopathological features of gastric cancer patients. POLE2 knockdown was induced, while POLE2 overexpression inhibited ferroptosis in human gastric cancer cells, thereby controlling the malignant phenotypes of gastric cancer. Mechanistic studies revealed that POLE2 overexpression elevated NRF2 expression and activity and subsequently activated GPX4, which then prevented ferroptosis of human gastric cancer cells. In contrast, either NRF2 or GPX4 silence significantly prevented POLE2 overexpression‐mediated induction of cell malignancies and inhibition of ferroptosis. In general, our findings first reveal that POLE2 overexpression inhibits ferroptosis of human gastric cancer cells through activating the NRF2/GPX4 pathway and provide a theoretical principal of molecular therapy for gastric cancer through inhibiting POLE2.

Ferroptosis, featured as iron relied overproductions of lipid ROS, is a newly identified form of regulated cell death and differs from the traditional programmed cell death. Iron overload contributes to executing ferroptosis and donates electrons to oxygen to produce toxic L‐OOH from the PUFAs, which in turn initiate and propagate oxidative PUFAs fragmentation and membrane destruction.^9^, ^49^ In the existence of iron, L‐OOH is converted into highly reactive lipid alkoxy radicals (L‐O·) that abstract protons from adjacent PUFAs and triggers continuous lipid peroxidation and further extension of oxidative damage.^50^ Accordingly, Tang et al. recently found that inducing iron accumulation significantly promoted lipid peroxidation and ferroptosis in human lung cancer cells.^29^ Yet, we herein found no alteration of intracellular iron level in human gastric cancer cells with POLE2 knockdown or overexpression. As a GSH‐associated antioxidase, GPX4 impedes the formation of L‐O· from L‐OOH and reduces L‐OOH to lipid alcohols, eventually suppressing PUFAs oxidation and ferroptosis.^12^ Cells, especially cancer cells with high metabolic rates, appear to continually suffer the threat of lipid ROS, and inhibiting GPX4 causes rapid accumulation of lipid ROS and oxidative damage.^12^ In addition, GPX4 deletion in mice is embryonic lethal.^51^ Furthermore, inhibiting GPX4 can sensitise cancer cells to ferroptosis and effectively restrain the development of human cancers.^52^ NRF2 can regulate lipid peroxidation and ferroptosis through regulating GPX4. Wang et al.^48^, ^53^, ^54^ revealed that inhibition of the NRF2/GPX4 pathway could sensitise colorectal cancer, non‐small cell lung cancer and hepatocellular carcinoma cells to ferroptosis. At this time, we determined that POLE2 knockdown inhibited, while POLE2 overexpression activated the NRF2/GPX4 pathway in human gastric cancer cells, and that either NRF2 or GPX4 silence blocked POLE2 overexpression‐mediated inductions of cell proliferation, migration, invasion and inhibition of ferroptosis. In addition, we found that POLE2 expression was induced by ferroptotic stimulation. As we know, POLE2 is a subunit of the DNA polymerase epsilon and plays critical roles in regulating DNA replication and repair. Ferroptosis is associated with the accumulation of toxic lipid ROS, which might result in oxidative damage to DNA and subsequently provoke DNA replication and repair. The activation of DNA replication and repair may elevate the expression of POLE2. Yet, the specific molecular basis mediating POLE2 elevation by ferroptotic stimuli should be determined in further study. In our study, we found that POLE2 expression was elevated in human gastric cancer tissues and cells and that POLE2 knockdown significantly induced ferroptosis of gastric cancer cells, thereby preventing the progression of gastric cancer. Given the role of POLE2 in gastric cancer and other tumours, we speculated that developing pharmacological inhibitors or neutralising antibody target for POLE2 would be of great therapeutic interest to treat human gastric cancer.

In general, our findings reveal that POLE2 overexpression inhibits ferroptosis of human gastric cancer cells through activating the NRF2/GPX4 pathway and provide a theoretical basis of molecular therapy for gastric cancer through inhibiting POLE2.

## AUTHOR CONTRIBUTIONS

**Hui Jian:** Conceptualization (equal); data curation (equal); investigation (equal); methodology (equal); validation (equal); visualization (equal); writing – original draft (equal); writing – review and editing (equal). **Zhi‐Qiang Chen:** Methodology (equal); project administration (equal); software (equal); validation (equal); visualization (equal). **Yang Yu:** Methodology (equal); project administration (equal); resources (equal); software (equal); supervision (equal); validation (equal); visualization (equal). **Heng Du:** Methodology (equal); resources (equal); software (equal); validation (equal); visualization (equal). **Ting Liao:** Methodology (equal); validation (equal); visualization (equal). **Yi‐Chen Sun:** Conceptualization (equal); investigation (equal); supervision (equal); visualization (equal); writing – original draft (equal); writing – review and editing (equal). **Dong Ke:** Methodology (equal); validation (equal); visualization (equal); writing – review and editing (equal).

## FUNDING INFORMATION

This work was supported by the Scientific Research Project of Hubei Provincial Health Commission (No. WJ2023M136) and Scientific Research Foundation of Jianghan University (No. 2023KJZXB02).

## CONFLICT OF INTEREST STATEMENT

The authors declare no conflicts of interest.

## Supporting information


Figures S1–S3.

## Data Availability

All data that support the findings in this study are available from the corresponding author upon reasonable request.
