# Supplementary material for: Inhibition of ferroptosis by POLE2 in gastric cancer cells involves the activation of NRF2/GPX4 pathway
Source: J Cell Mol Med. 2023 Dec 9;28(1):e17983. doi: 10.1111/jcmm.17983 (PMC10805511; doi:10.1111/jcmm.17983)
Supplement: Supplementary file 1 — Figures S1–S3. [file JCMM-28-e17983-s001.docx]

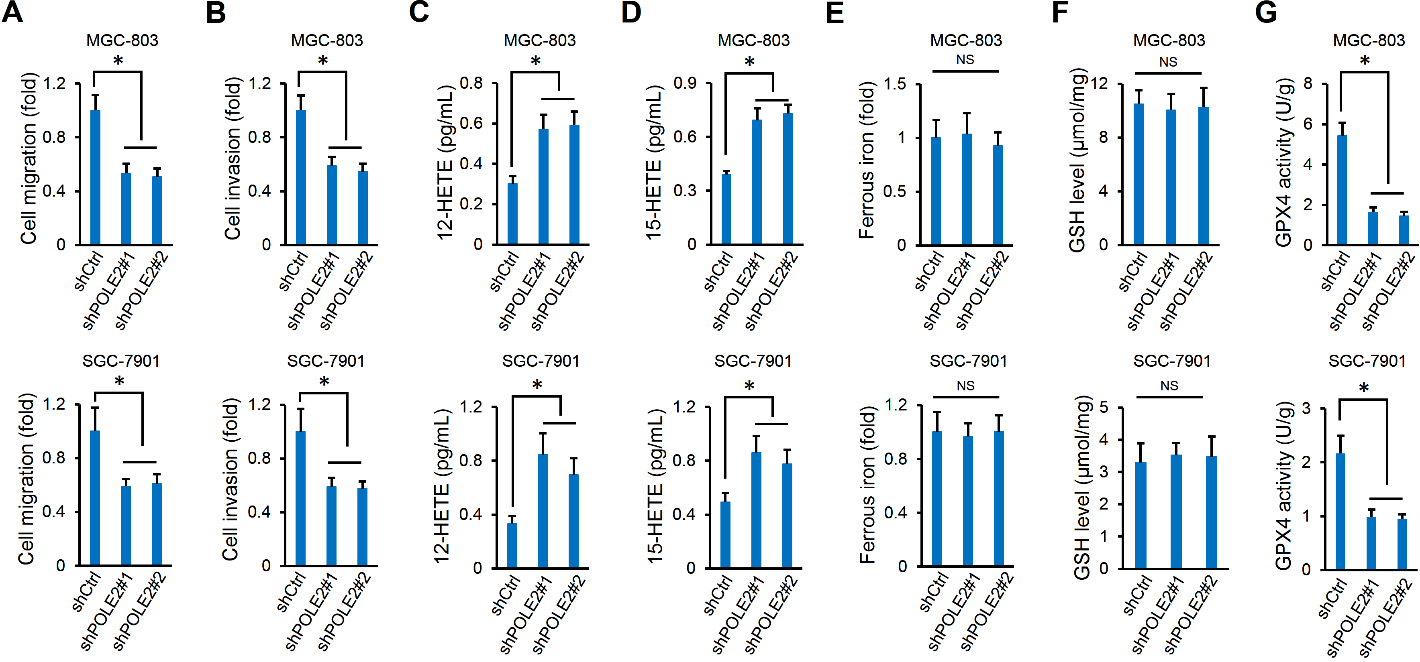


Figure S1. POLE2 knockdown inhibits the malignances of human gastric cancer cells. (A-B) Cell migration and invasion detected by Transwell assay. (C-D) 12/15-HETE levels in the medium. (E) Ferrous iron levels. (F) Intracellular GSH levels. (G) GPX4 activity levels. NS indicates no significance. n=6 and **P* < 0.05 versus matched groups.


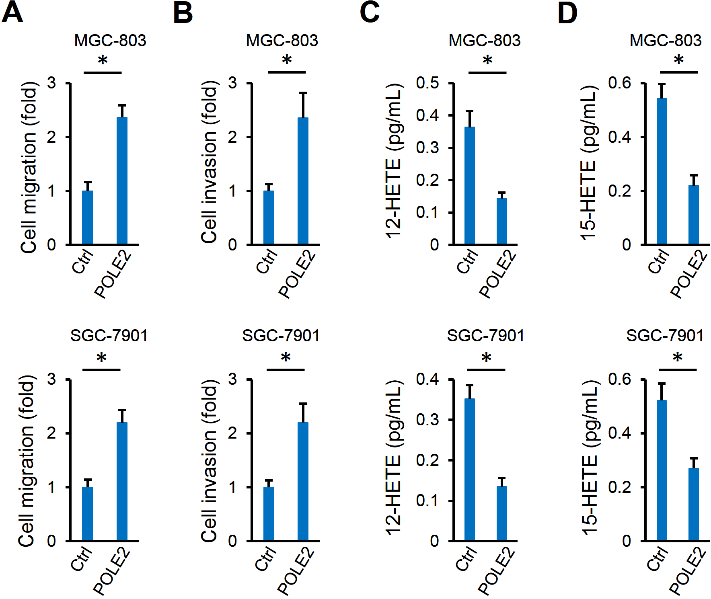


Figure S2. POLE2 overexpression facilitates the malignances of human gastric cancer cells. (A-B) Cell migration and invasion detected by Transwell assay. (C-D) 12/15-HETE levels in the medium. n=6 and **P* < 0.05 versus matched groups.


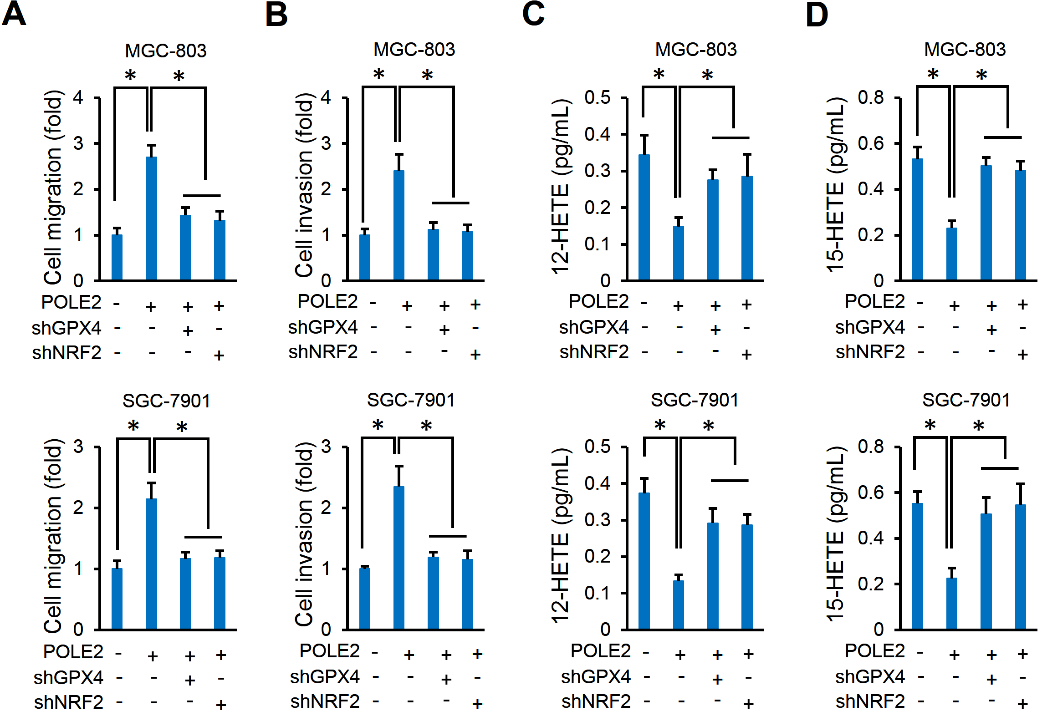


Figure S3. POLE2 overexpression inhibits ferroptosis of human gastric cancer cells through activating NRF2/GPX4 pathway. (A-B) Cell migration and invasion detected by Transwell assay. (C-D) 12/15-HETE levels in the medium. n=6 and **P* < 0.05 versus matched groups.
